# Nanograting p-n Junctions with Enhanced Charge Confinement

**DOI:** 10.3390/nano14231889

**Published:** 2024-11-24

**Authors:** Avtandil Tavkhelidze, Larisa Jangidze, Givi Skhiladze, Sergo Sikharulidze, Kristine Dzneladze, Rusudan Kvesitadze, Amiran Bibilashvili

**Affiliations:** 1 Center of Nanotechnology for Renewable Energy, Ilia State University, Cholokashvili Ave. 3/5, Tbilisi 0162, Georgia; larisa.jangidze@iliauni.edu.ge (L.J.); kristine.dzneladze.1@iliauni.edu.ge (K.D.); rusudan.kvesitadze.1@iliauni.edu.ge (R.K.); 2 Institute of Micro and Nano Electronics, Chavchavadze Ave. 13, Tbilisi 0179, Georgiaamiran.bibilashvili@tsu.ge (A.B.)

**Keywords:** nanograting, p-n junction, exciton, solar cell, flat band

## Abstract

Recently, geometry-induced quantum effects in a new quasi-1D system, or nanograting (NG) layers, were introduced and investigated. Dramatic changes in band structure and unconventional photoluminescence effects were found in silicon quantum wells with high-energy barriers. Nanograting metal–semiconductor junctions were fabricated and investigated. Here, we report the latest results on a special type of p-n junction in which the charge confinement of the NG is enhanced. The reverse bias dark current is increased in contrast to the metal–semiconductor junctions. When such a junction works as a photovoltaic cell, NG significantly increases short-circuit current and conversion efficiency without affecting open-circuit voltage. These effects are explained by the formation of geometry-induced excitons. To distinguish exciton formation from G-doping effects, we fabricated NGs in both n-type and p-type top layers and obtained qualitatively the same results. To further verify the excitonic mechanism, we analyzed photoluminescence spectrums previously obtained from NG and other NG-like periodic structures. The collected experimental results and previous findings are well explained by the formation of geometry-induced excitons and corresponding quasi-flat bands. Geometry-induced quantum effects can be used to significantly increase the conversion efficiency of photovoltaic cells and enhance the characteristics of other optoelectronic devices.

## 1. Introduction

The latest developments in nanotechnology have allowed for the fabrication of low-dimensional periodic nanostructures [1,2,3,4] including nanogratings (NGs). Imposed periodic nanostructures such as NGs are known to significantly affect the electronic [5,6,7,8,9,10,11], optical [12,13,14,15,16,17,18,19] and other properties of semiconductors when the NG depth becomes comparable to the de Broglie wavelength  λ. Nanograting forms the layer regarded as the quasi-1D structure with reduced DOS. Inside such a layer, valence-band electrons have to transfer to the conduction band, a process termed geometry-induced doping or G-doping [7]. Experiments conducted on NG layers confirmed dramatic changes in optical properties. Ellipsometry measurements showed dramatic changes in the interband density of states as new peaks emerged and conventional ones disappeared [12]. Photoluminescence spectroscopy revealed equidistant peaks in the energy range of 2–3 eV [12,17]. These changes were not reliably explained. Later, similar PL spectrums were recorded from the quasi-1D van der Waals semiconductor flake with grating-like geometry [13] and from the InGaN/GaN grating-like structure [14]. Analogous PL spectrums were recorded from bilayer graphene and other materials at low temperatures and were explained by the formation of moire excitons and corresponding quasi-flat bands [20,21,22,23,24,25,26]. Based on the similarity of the PL spectrums, we consider the NG layer a structure that hosts excitons. In fact, NG imposes periodic potential in the X-direction, generating periodic patterns and excitons in the periodic potential formed. Excitons were studied in other low-dimensional structures as well [27,28]. 

**Figure 1:**
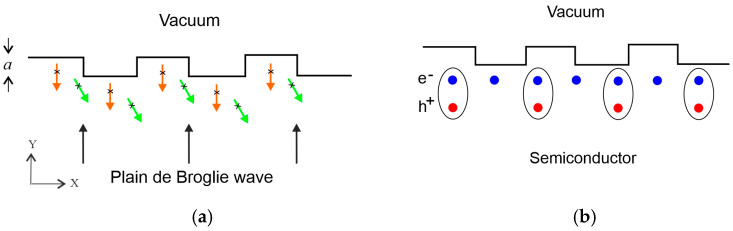
(**a**) Electron reflection and (**b**) geometry-induced charge accumulation and exciton formation at the nanograting layer. Red arrows depict a plain de Broglie wave moving towards the NG surface of the semiconductor. Orange nd green arrows depict waves reflected from the top and bottom of the indents. Electrons are depicted by blue dots and holes by red. Ellipse depict an exciton.

Figure 1a shows NG fabricated on the surface of the semiconductor and a plain de Broglie wave propagating towards the surface (black arrows). It reflects partly from the indents and partly from the protrusions (orange arrows). Two waves will interfere destructively when $\lambda_{n}=2a/(n+1/2)$, where *a* is the depth of the indent and n is an integer. Consequently, electrons with wavelengths $\lambda_{n}\;$ cannot reflect back from the NG. It seems that electrons can reflect back from the NG at different angles by which the destructive interference turns into the constructive one (green arrows). However, such reflection requires changing wave vector $\vec{k}$ in the process of reflection and is quantum mechanically forbidden as there is a Fermi gas inside the semiconductor (all quantum states are already occupied). Lastly, electrons cannot penetrate the vacuum as they do not have enough energy to overcome the energy barrier. Hence, all possible final quantum states are forbidden for electrons with initial wave vector Y-components $k_{n}=2\pi /\lambda_{n}$. Consequently, DOS reduces [29] and semiconductor properties change. These changes were confirmed experimentally [5,6,12,17,18] and investigated theoretically [7,29].

G-doping requires energy that is more than the band gap for transferring valence-band electrons to the conduction band (at T = 0). The alternative mechanism, which can have a lower energy cost, is the movement of the electrons (Figure 1b) near the NG (instead of reflecting back from the NG). In this process, negative charge will collect near the NG which will also increase system energy by the amount of $eV_{NG},\;$ (per electron), where $V_{NG}$ is the electric potential of the NG relative to the bulk semiconductor. Accumulation of negative charge will be energetically advantageous until the energy needed for adding one more electron to the NG area $eV_{NG}<(3/5)\Delta E_{F},\;$ where $\Delta E_{F}\;$ is the increase in Fermi energy owing to G-doping (coefficient 3/5 comes from the average energy of electrons in Fermi gas). Further, excitons can form to reduce system energy. The electron rejected from the forbidden quantum state can form an exciton instead of occupying a high-energy level (G-doping) or adding charge to the NG. During exciton formation, an electron leaves the initial Fermi–Dirac distribution ($\Delta E_{F}$ = 0) and becomes a Boson together with a hole. Dissociation of the exciton returns the electron to the final Fermi–Dirac distribution with $\Delta E_{F}>0\;$ at a higher energy level with extra energy. Consequently, more energy is needed to dissociate the exciton near the NG. In other words, G-doping increases the average energy of electrons, which makes exciton dissociation difficult. Our previous experiments show that $\Delta E_{F}$ can be as high as 0.2 eV [30] giving $(3/5)\Delta E_{F}$ = 0.12 eV. This is considerably more than thermal energy KT = 0.026 eV at room temperature where excitons stabilize near the NG. Other known mechanisms of exciton formation at the surface are dielectric disorder [31] and a large number of lattice defects (room temperature) [32]. 

Nanograting layers have obvious advantages over mono- and bilayers in terms of charge carrier transport and light confinement. The thickness is 100–300 nm, which is 2–3 orders of magnitude greater than mono- and bilayers. Nanograting layers can be directly embedded in the p-n junction as their thickness is of the order of the typical charge depletion region. Adding an NG layer to the solar cell p-n junction can actually increase light absorption by confining light and effectively converting photons to electron–hole pairs.

Geometry-induced quantum effects strongly depend on the energy barrier at the NG layer. Actually, it is more energetically advantageous for the electron to overcome a low-energy barrier than to transfer from the valence band to the conduction band (G-doping). Consequently, a high-energy barrier is needed to obtain distinct geometry-induced quantum effects. In our previous experiments with metal–semiconductor junctions [33], conventional metal contacts were used, and the energy barrier formed between the semiconductor and metal was roughly 0.2 eV, which is less than the Si band gap. Here, we utilized a special type of p-n junction with a grid-type front metal contact structure usually used for solar cells. Such contact allows for the preservation of the high-energy barrier (>4 eV) at most parts of the NG surface and, at the same time, collects charge carriers from it. To distinguish changes introduced by geometry-induced excitons from G-doping effects, we performed G-doping in both n-type and p-type top layers and compared the results.

## 2. Materials and Methods

Two Si substrates of different types were used in this study: the p-type 1–5 Ω × cm corresponding to hole concentration p = 3 × 10^15^–2 × 10^16^ cm^−3^ and n-type 1–10 Ω × cm corresponding to electron concentration n = 5 × 10^14^–5 × 10^15^ cm^−3^. First, the samples were cut into chips with dimensions of 10 mm × 20 mm. Next, two 6 mm × 6 mm area p-n junctions were formed in the centers of 10 × 10 mm half parts using typical thermal diffusion technology.

**Figure 2:**
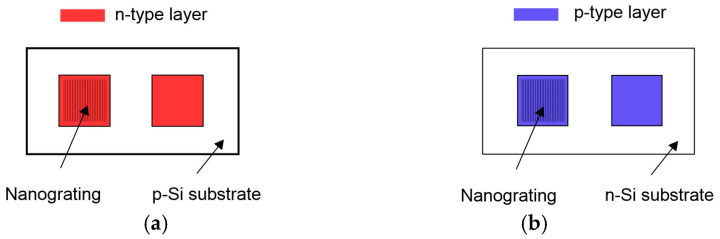
NG sample layout: (**a**) n-type top layer formed in p-type substrate; (**b**) p-type top layer formed in n-type substrate. The front ohmic contact grid is omitted for simplicity.

For p-type substrates (Figure 2a), boron was diffused in a depth of 0.6–0.7μ from the back side at 1100 °C in the Ar atmosphere for 5 min. Next, phosphorus was diffused in a depth of 0.3–0.4 μ from the front side at 1050 °C in the Ar atmosphere for 3 min. The 1 μ thick Al film was deposited from the back side and baked at 500 °C for 15 min. 

For n-type substrates (Figure 2b), phosphorus was diffused in a depth of 0.6–0.7 μ from the back side at 1050 °C in the Ar atmosphere for 5 min. Next, boron was diffused in a depth of 0.3–0.4 μ from the front side at 1100 °C in the Ar atmosphere for 3 min. The 2 μ thick In film was deposited from the back side and baked at 100 °C for 20 min. 

An NG layer with a linewidth of 150 nm and indent depth *a* = 20 nm was fabricated inside the top layer over an area of 5 mm × 5 mm (at the center of the half chip) by laser interference lithography, followed by reactive-ion etching (RIE) [5]. A negative photoresist was employed during lithography to ensure even etching of the entire chip’s surface, including the reference area, as in previous works [5,18]. This step was taken to eliminate the influence of RIE during comparison with the reference plain area. The surface roughness measured after RIE did not exceed RMS = 1 nm.

**Figure 3:**
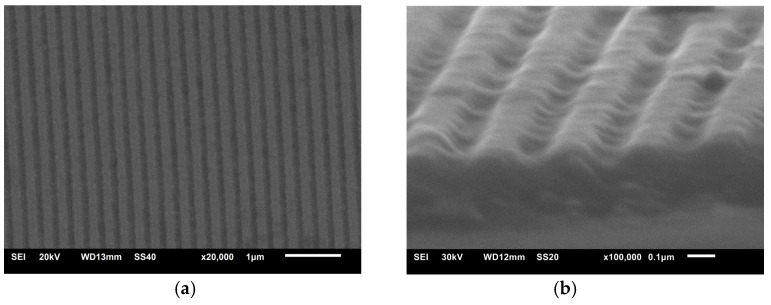
SEM images of the nanograting: (**a**) large scale indicating homogeneity and (**b**) small scale representing surface morphology.

Scans of the electron microscope images of the NG are shown in Figure 3. The NG cross-section is closer to the wave which is typical for laser interference lithography.

**Figure 4:**
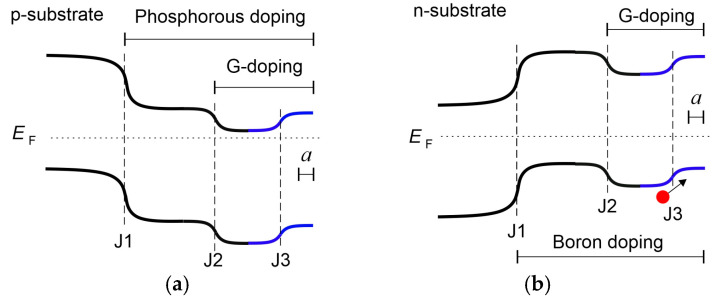
Energy diagrams of Si solar cell p-n junctions: (**a**) G-doped top layer induced in n-type layer formed by phosphorus diffusion in p-type substrate; (**b**) G-doped top layer induced in p-type layer formed in n-type substrate by boron diffusion. Nanograting depth *a* is indicated for depth scale estimation. The direction of the incident light is from right to left (hits junctions in sequence J3, J2, and J1). Blue color depicts potential profile of Junction 3. Red dot depicts a hole.

The 0.8 μ thick Al film was deposited from the front side and baked at 200 °C for 30 min. Photolithography was used to shape the Al grid consisting of 12 lines with a length of 5 mm and a width of 0.04 mm arranged in a conventional solar cell grid shape. Each nanograting p-n junction was prepared in pairs with a plain reference p-n junction (placed at the same 10 × 20 mm chip) to reveal the impact of the NG. Nanograting introduced G-doping in both n-type (Figure 4a) and p-type (Figure 4b) top layers. G-doping is n-type by its nature and consequently leads to the downward bending of the bands in both cases. On the contrary, negative charge accumulation at the NG introduces upward bending of bands in both cases and is depicted by a blue color in the energy diagrams shown in Figure 4. There are three junctions in both types of samples. The first junction (J1 in Figure 3) is a conventional p-n junction formed in the process of dopant (phosphorous or boron) diffusion. The second junction (J2) is formed in the process of NG fabrication and is a G-doped junction embedded in the n-type layer (Figure 4a) or in the p-type layer (Figure 4b). G-doped junction causes downward bending of band edges (as G-doping is n-type) and has a depth of 0.1–0.2 μ. The third junction (J3) is formed in the process of electron accumulation near the NG (Figure 1b). Junction J3 causes upward bending of band edges as it is produced by negative charge accumulation. The direction of the incident light is from right to left.

The parameters of p-n junctions and metal contacts were chosen to investigate optical properties and were not optimized for maximum power generation (also there was no anti-reflection layer).

The dark I-V and illuminated I-V and P-V characteristics of junctions were measured. A Keysight multimeter 34410, current source E3640, solar radiation simulator (ABET Technologies Inc., Milford, CT, USA Model 10500) with spectrum AM1.5G, and homemade LabVIEW drivers were used to record I-V and P-V curves.

## 3. Results

To collect all evidence of excitons in NG layers in this section we (a) present the results of I-V and P-V characteristics recorded from specially designed nanograting p-n junctions and compare them to their plain counterparts and (b) compare PL spectra presented in our previous and more recent works from the literature related to nanograting-like structures and also with spectrums from bilayer structures.

### 3.1. Solar Cell-like p-n Junctionours

**Figure 5:**
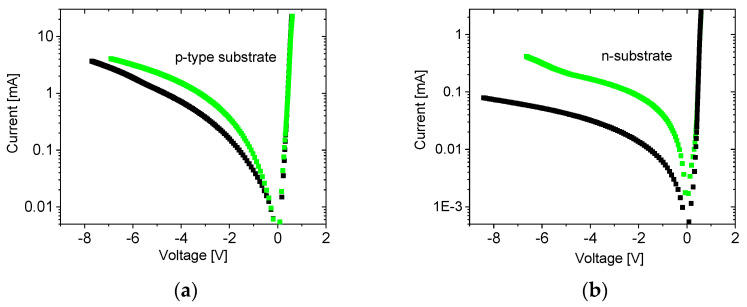
Dark I-V characteristics of p-n junctions: (**a**) G-doped layer induced in n-type layer formed in p-type substrate; (**b**) G-doped layer induced in p-type layer formed in n-type substrate. Green G-doped junction, black corresponding reference plain junction.

Figure 5 shows typical dark I-V characteristics of nanograting p-n junctions formed on p-type (a) and n-type (b) substrates. There is no notable difference between the forward bias characteristics. The reverse current of the NG junction (green) is higher than for the plain reference junction (black) for both p-substrate and n-substrate junctions. However, the increase in reverse current by the nanograting is much more pronounced for the n-substrate junction. 

**Figure 6:**
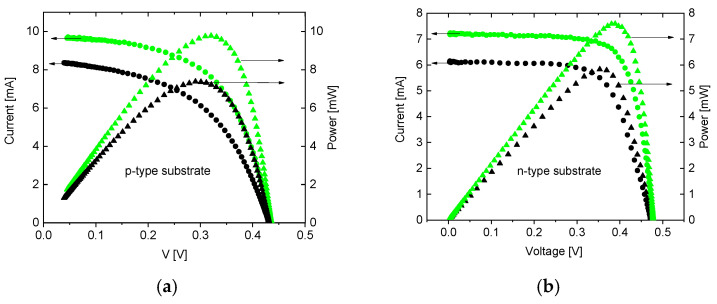
Illuminated I-V and P-V characteristics of photovoltaic cells: (**a**) G-doped layer induced in n type layer formed in p-type substrate; (**b**) G-doped layer induced in p-type layer formed in n-type substrate. Green depicts G-doped junction, black color depicts reference plain junction.

Figure 6 shows typical illuminated (spectrum AM1.5G) I-V and P-V characteristics of p–n junctions in photovoltaic conversion mode. It is obvious that G-doping increases short-circuit current $I_{sc}$ and maximum power output $P_{max}$ for both types of junctions. At the same time, open-circuit voltage $V_{oc}$ remains unaffected. Overall, qualitative changes introduced by the G-doping are independent of the type of layer in which it was realized (NG is formed).

### 3.2. Photoluminescence from Nanograting Layers

Direct observation of exciton peaks in the photoluminescence spectrum is not possible at room temperature as exciton binding energy is only 10 meV in Si material (low compared to thermal energy KT = 26 meV). At the same time, our p-n junction samples operate only at room temperature as cooling down will freeze out charge carriers. The following calculations were made to evaluate the possibility of direct observation of geometry-induced exciton peaks: we took experimental values of $\Delta E_{F}$ = 0.15–0.2 eV from [30] and calculated effective temperature near the NG by comparison of $(3/5)\Delta E_{F}$ with KT. The obtained effective temperatures are 66 K for n-type and 83 K for p-type NGs. Corresponding thermal energies are 5.8 meV and 7.2 meV. These values are still enough to smooth exciton peaks. Despite the reduced effective temperature, direct observation of geometry-induced excitons remains challenging in Si material [34], especially in our case when we could not lower the sample temperature. Charge carriers will freeze out and the energy diagram will transform dramatically (p-n junction will disappear below 100 K). To magnify geometry-induced quantum effects, strong carrier confinement from both sides of the NG ensuring a higher value of $\Delta E_{F}$ is necessary. Unfortunately, this is not the case for our junctions. The energy barrier remains relatively low (about 0.5 eV) from the left side of the G-doped area (Figure 4) and $\Delta E_{F}$ < 0.2 eV.

For this reason, we had to return to our previous results obtained from SOI-thin NG layers. In that system (with barriers > 4 eV from both sides and $\Delta E_{F}$ > 0.5 eV), we obtained a wide PL emission peak positioned at energies 2–4 eV and superimposed equidistant peaks [12,17]. Remarkably, such peaks have not been obtained by other authors (at room temperature) until recently when it was reported in two works studying structures with NG-like geometry: (a) 1D van der Waals semiconductor flake [13] and (b) InGaN/GaN quantum structures [14]. In all three works [13,14,17], equidistant PL peaks were recorded from NG or NG-like geometries. Equidistant peak separation varies between 80 and 130 meV. All three structures are made from different semiconductor materials, but their dimensions (indent depth and indent periods) are similar to each other.

To find a reasonable explanation, we performed an intensive study of the literature and found a similar PL spectrum emitted by twisted bilayer WS_2_ at low temperatures [20]. This structure has periodic potential (moire pattern) and hosts a spatial type of exciton named moire excitons which create flat excitonic minibands. Comparable spectrums were recorded from other twisted bilayer structures [20,21,22,23,24,25]. The similarity of PL spectrums indicates that our nanograting SOI samples also host excitons in a periodic potential. Consequently, we can expect localized excitons in our nanograting p-n junction samples as well.

## 4. Discussion

Figure 5 indicates that NG increases the reverse bias dark current. This cannot be explained by G-doping as it reduces the reverse current. This effect was confirmed by experiments on an NG metal–semiconductor junction [33]. A possible explanation is the formation of localized excitons near the NG. Excitons can be dissociated by a strong electric field when a reverse voltage is applied. Dissociated excitons generate additional electrons and holes and can increase reverse current. The positive effect of the localized excitons seems to be superior as device performance is not compromised by the increase in reverse current. Such an increase indicates a rise in leakage current which should reduce both $V_{oc}$ and $I_{sc}$. However, as Figure 6 shows $V_{oc}$ does not change and $I_{sc}$ is even improved.

Figure 6 indicates that NG increases the maximum output of power $P_{max}$ and short-circuit current $I_{sc}$, not affecting open-circuit voltage $V_{oc}$. This corresponds to an increase in photocurrent by increasing the absorption of photons. The formation of excitons near the NG explains these results quite well. Excitons absorb photons at the surface. Further, excitons migrate to the p-n junction area and dissociate inside the depletion layer. This process generates additional charge carriers which are spatially separated by built-in potential and $I_{sc}$ increases. This is consistent with measurements of quantum efficiency which show that NG increases light absorption [18].

Both the increase in reverse current (Figure 5) and the increase in $I_{sc}$ (Figure 6) happen independently of the type (n or p) of the layer in which NG was formed. This fact also supports the exciton formation mechanism. Indeed, G-doping can create only free electrons and is n-type by its nature. Consequently, different results are expected depending on the type of layer in which NG is fabricated. However, we obtained quantitatively the same result in both n-type and p-type doping of the initial top layer. This indicates that an increase in reverse current and $I_{sc}$ does not originate from G-doping and is a result of an extra mechanism (exciton formation). 

The geometry-induced excitonic mechanism of increasing $I_{sc}$ is also supported by increasing the fill factor (and consequently photocurrent) for the initially p-type nanograting layer (Figure 6b) with respect to the initially n-type layer (Figure 6a). It can be explained by majority carrier confinement to the NG. This additional confinement is caused by the original confinement of electrons to the NG and the corresponding upward winding of the valence band maximum (Figure 4b, blue). Such confinement of holes to the NG further increases exciton concentration and the resulting fill factor, with respect to the n-type initial layer.

The extraordinary photoluminescence spectrum of NG layers can be explained by localized exciton formation. We have an obvious analogy between the PL spectrum of NG layers and spectrums of moire excitons in twisted bilayer structures. The spectrum contains wide peaks with superimposed equidistant peaks which are authentic for excitons in periodic potential. This is also supported by the fact that NG is creating periodic 2D potential in the X–Y plane (Figure 1a). PL emission from twisted bilayers is anisotropic [20,21,22,23,24,25]. It is anisotropic for NG-like structures as well [13,14]. Finally, we also measured anisotropic PL from our NG layers. PL emission anisotropy can be explained by the fact that NG layers have quantum properties in the X–Y plane and do not have them along the Z-axis.

Generally, incident light trapping by the NG can contribute to an increase in light absorption and an increase in $I_{sc}$. The indent depth is 20 nm which is much less when compared to the shortest wavelength (250 nm) of the solar spectrum. For comparison, textured Si solar cell surfaces optimized for light absorption [35,36] have a periodicity of 3–5 μ (which is 10–15 times more than our NG period) and pyramid height of 2 μ (which is 100 times more than our NG indent depth). Consequently, light trapping by the NG can be ignored in the first approximation (mostly because of light diffraction on ridges). Localized surface plasmons can also form at the interface of the Al grid and NG and contribute to $I_{sc}$. Localized plasmon area is estimated as 0.5 μ (average light wavelength) multiplied by the total length of the Al wires is 60 mm and factor 2, resulting in 6 × 10^−4^ cm^2^. This is almost three orders of magnitude less than the total area (0.25 cm^2^), and consequently, localized plasmon contribution can be ignored. Obviously, light trapping and localized plasmons cannot increase the dark reverse current (Figure 5).

Care should be taken when considering excitons at room temperature. Generally, exciton binding energy should be more than thermal energy to obtain a long lifetime of the quasiparticle. However, there are at least two known mechanisms, namely dielectric constant variation and concentrated defects, which extend exciton lifetime near the surfaces. Dielectric constant variation originates from spatial fluctuations of the surface morphology and polarizable impurities [31]. Concentrated defects are consequence of the crystal-surface-behavior-like lattice defects tending to host excitons [31,37,38]. The last mechanism works even at room temperature. By suggesting a new exciton formation mechanism (geometry-induced), we extend the list of surface exciton types.

Geometry-induced exciton formation at nanograting surface can be utilized for increasing light absorption and carrier generation in p-n junctions and consequent increase in solar cell conversion efficiency.

## 5. Conclusions

Nanograting p-n junction photovoltaic cells of different types were fabricated and investigated. Nanograting was formed in p-type and n-type top layers and I-V and P-V characteristics were recorded for comparison. In both types of p-n junctions, nanograting increases reverse bias dark current. Nanograting increases short-circuit current and conversion efficiency without affecting open-circuit voltage in the process of photovoltaic conversion. These effects are explained by geometry-induced exciton formation near the nanograting. Exciton formation is energetically advantageous as it neutralizes localized negative charge and reduces system energy. When a reverse bias is applied, the electric field dissociates excitons and generates additional charge carriers, increasing dark reverse current. During photovoltaic conversion, photons are absorbed by the excitons diffusing towards the depletion layer where they dissociate and additional photocurrent is generated. To verify the excitonic mechanism, the photoluminescence spectrum previously obtained from the nanograting layers was compared to spectrums recorded from other nanograting-like structures and from the twisted bilayers. Nanograting layers hosting excitons can be utilized to increase solar cell efficiency and improve the characteristics of other photovoltaic devices.

## Data Availability

No research data to share.

## References

[1] Liu R., Cao L., Liu D., Wang L., Saeed S., Wang Z. (2023). Laser Interference Lithography—A Method for the Fabrication of Controlled Periodic Structures. Nanomaterials.

[2] Lee S., Ma Y., Park J., Shin B. (2021). Fabrication of uniform diffraction gratings using laser interference lithography for simultaneous measurement of refractive index. Jpn. J. Appl. Phys..

[3] Buhl J., Yoo D., Köpke M., Gerken M. (2021). Two-Dimensional Nanograting Fabrication by Multistep Nanoimprint Lithography and Ion Beam Etching. Nanomanufacturing.

[4] Bashirpour M., Cui W., Gamouras A., Ménard J.-M. (2022). Scalable Fabrication of Nanogratings on GaP for Efficient Diffraction of Near-Infrared Pulses and Enhanced Terahertz Generation by Optical Rectification. Crystals.

[5] Tavkhelidze A., Jangidze L., Mebonia M., Piotrowski K., Więckowski J., Taliashvili Z., Skhiladze G., Nadaraia L. (2017). Geometry-induced quantum effects in periodic nanostructures. Phys. Status Solidi (a).

[6] Liang W., Meng S., Zeng Q., Han X. (2022). Intrinsic Connections between Thermionic Emission Cooling Effect and Emission Characteristics of W-La_2_O_3_ Cathodes at High Temperatures. Mater. Lett..

[7] Tavkhelidze A. (2014). Geometry-induced electron doping in periodic semiconductor nanostructures. Phys. E.

[8] Chae M., Kim S., Han Y., Choi D., Choi Y., Kim H., Joo M.K. (2022). High Temperature Carrier Scattering Mechanisms in Multilayer ReS_2_ Field-Effect Transistors. Appl. Sci. Converg. Technol..

[9] Wang Q., Tang Y., Miura A., Miyazaki K., Horita Z., Iikubo S. (2024). Improving Thermoelectric Properties of Bi_2_Te_3_ by Straining under High Pressure: Experiment and DFT Calculation. Scr. Mater..

[10] Mamedov N., Tavkhelidze A., Bayramov A., Akhmedova K., Aliyeva Y., Eyyubov G., Jangidze L., Skhiladze G. (2017). Spectroscopic planar diffraction ellipsometry of Si-based multilayer structure with subwavelength grating. Phys. Status Solidi C.

[11] Singh A.K., Kumar J. (2021). Nano-gap planar metal electrodes: Fabrication and I–V characteristics. Nano Express.

[12] Bayramov A., Alizade E., Mammadov S., Tavkhelidze A., Mamedov N., Aliyeva Y., Ahmedova K., Asadullayeva S., Jangidze L., Skhiladze G. (2019). Optical properties of surface grated Si-based multilayer structure. J. Vac. Sci. Technol. B.

[13] Du L., Zhao Y., Wu L., Hu X., Yao L., Wang Y., Bai X., Dai Y., Qiao J., Uddin G. (2021). Giant anisotropic photonics in the 1D van der Waals semiconductor fibrous red phosphorus. Nat. Commun..

[14] Yeo H.-S., Lee K., Sim Y.C., Park S.-H., Cho Y.-H. (2020). Strong and robust polarization anisotropy of site- and size-controlled single InGaN/GaN quantum wires. Sci. Rep..

[15] Fang Z., Zhao Y., Shao J. (2016). Femtosecond Laser-Induced Periodic Surface Structure on Fused Silica Surface. Optik.

[16] Pérez M., Ramos E., de la Mora M., Santana G., Dutt A. (2021). Absorption and Emission of Porous Silicon Based on Quantum Dots Models by TD-DFT: Experimental and Theoretical Approach. Mater. Lett..

[17] Tavkhelidze A., Bayramov A., Aliyeva Y., Jangidze L., Skhiladze G., Asadullayeva S., Alekperov O., Mamedov N. (2017). Optical and electronic properties of periodic Si nanostructures. Phys. Status Solidi C.

[18] Taliashvili Z., Łusakowska E., Chusnutdinow S., Tavkhelidze A., Jangidze L., Sikharulidze S., Gorji N.E., Chubinidze Z., Melkadze R. (2023). Optical Properties of Periodically and Aperiodically Nanostructured p-n Junctions. Opt. Quantum Electron..

[19] Mastellone M., Pace M.L., Curcio M., Caggiano N., De Bonis A., Teghil R., Dolce P., Mollica D., Orlando S., Santagata A. (2022). LIPSS Applied to Wide Bandgap Semiconductors and Dielectrics: Assessment and Future Perspectives. Materials.

[20] Wu B., Zheng H., Li S., Ding J., Zeng Y.-J., Liu Z., Liu Y. (2022). Observation of Moiré Excitons in the Twisted WS2/WS2 Homostructure. Nanoscale.

[21] Rodríguez Á., Varillas J., Haider G., Kalbáč M., Frank O. (2023). Complex Strain Scapes in Reconstructed Transition-Metal Dichalcogenide Moiré Superlattices. ACS Nano.

[22] Liu Y., Zeng C., Yu J., Zhong J., Li B., Zhang Z., Liu Z., Wang Z.M., Pan A., Duan X. (2021). Moiré Superlattices and Related Moiré Excitons in Twisted van der Waals Heterostructures. Chem. Soc. Rev..

[23] Tran K., Moody G., Wu F., Lu X., Choi J., Kim K., Rai A., Sanchez D.A., Quan J., Singh A. (2019). Evidence for moiré excitons in van der Waals heterostructures. Nature.

[24] Zheng H., Wu B., Li S., He J., Chen K., Liu Z., Liu Y. (2022). Evidence for interlayer coupling and moiré excitons in twisted WS2/WS2 homostructure superlattices. Nano Res..

[25] Zheng H., Wu B., Li S., Ding J., He J., Liu Z., Wang C.-T., Wang J.-T., Pan A., Liu Y. (2023). Localization-enhanced moiré exciton in twisted transition metal dichalcogenide heterotrilayer superlattices. Light Sci. Appl..

[26] Adler E.R., Le T.D.M., Boulares I., Boyd R., He Y., Rhodes D., Van Keuren E., Barbara P., Najmaei S. (2024). Observation of Multi-Phonon Emission in Monolayer WS_2_ on Various Substrates. Nanomaterials.

[27] Wu S., Tomić S. (2012). Exciton States and Oscillator Strengths in a Cylindrical Quantum Wire with Finite Potential under Traverse Electric Field. J. Appl. Phys..

[28] Blancon J.-C., Stier A.V., Tsai H., Nie W., Stoumpos C.C., Traoré B., Pedesseau L., Kepenekian M., Katsutani F., Noe G.T. (2018). Scaling law for excitons in 2D perovskite quantum wells. Nat. Commun..

[29] Kakulia D., Tavkhelidze A., Gogoberidze V., Mebonia M. (2016). Density of quantum states in quasi-1D layers. Phys. E.

[30] Tavkhelidze A., Bibilashvili A., Jangidze L., Gorji N.E. (2021). Fermi-Level Tuning of G-Doped Layers. Nanomaterials.

[31] Raja A., Waldecker L., Zipfel J., Cho Y., Brem S., Ziegler J.D., Kulig M., Taniguchi T., Watanabe K., Malic E. (2019). Dielectric disorder in two-dimensional materials. Nat. Nanotechnol..

[32] Wang J., Chen C.-X., Rao J.-R., Cao L., Ma G.-Z., Gao M., Chen X.-C., Shi D. (2022). Spectroscopic Indications of Room-Temperature Electron-Hole Droplets in Optically Excited CH3NH3PbBr3 Single Crystals. Cell Rep. Phys. Sci..

[33] Tavkhelidze A., Jangidze L., Taliashvili Z., Gorji N.E. (2021). G-Doping-Based Metal-Semiconductor Junction. Coatings.

[34] Revuelta S., Cánovas E. (2023). Exciton formation dynamics at the SiO_2_/Si interface. Commun. Mater..

[35] Saive R. (2021). Light Trapping in Thin Silicon Solar Cells: A Review on Fundamentals and Technologies. Prog. Photovolt. Res. Appl..

[36] Manzoor S., Filipič M., Onno A., Topič M., Holman Z.C. (2020). Visualizing Light Trapping within Textured Silicon Solar Cells. J. Appl. Phys..

[37] Gatos H.C. (1962). Crystalline Structure and Surface Reactivity: Atomistic models are unique tools for dealing with the chemical and physical properties of surfaces. Science.

[38] Saba M., Cadelano M., Marongiu D., Chen F., Sarritzu V., Sestu N., Figus C., Aresti M., Piras R.G., Lehmann A.G. (2014). Correlated Correlated electron-hole plasma in organometal perovskites. Nat. Commun..

